# The New Zealand podiatry profession – a workforce in crisis?

**DOI:** 10.1186/s13047-020-00430-y

**Published:** 2020-10-12

**Authors:** Matthew Carroll, Hannah Jepson, Prue Molyneux, Angela Brenton-Rule

**Affiliations:** Department of Podiatry, School of Clinical Sciences, Faculty of Health & Environmental Sciences, Auckland University of Technology, Private Bag 92006, Auckland, 1142 New Zealand

**Keywords:** New Zealand, Podiatry, Workforce

## Abstract

**Background:**

This is the first study to explore workforce data from the Podiatrists Board of New Zealand. The study analysed data from an online survey which New Zealand podiatrists complete as part of their application for an Annual Practising Certificate.

**Methods:**

Survey responses between 2015 and 2019 were analysed. Data was related to work setting, employment status, work hours, location, professional affiliations, and number of graduates entering practice. Survey data was downloaded by a second party who provide data security for the Podiatrists Board of New Zealand workforce data. All data supplied for analysis were deidentified and could not be re-linked to an individual practitioner.

**Results:**

In 2019 there were 430 podiatrists who held an Annual Practising Certificate. Eighty percent of podiatrists who work in New Zealand are in private practice, with 8% employed in the public health sector. Podiatrist’s work is a mix of general podiatry, diabetes care and sports medicine. The majority are self-employed (40%) or business owners (19%). Approximately 40% work between 31 to 40 h per week and 46 to 50 weeks per year. The majority are female (67%) with most practising in the North Island (69%) and located in the Auckland region (33%). On average 76% of new graduates were issued an Annual Practising Certificate between 2015 and 2019.

**Conclusion:**

The New Zealand podiatry profession is small and growing at a slow rate, consequently there is evidence of a workforce shortage. To maintain a per-capita ratio of podiatrists approximate to Australia and the United Kingdom an additional 578 podiatrists are required in the New Zealand workforce. There are not enough new graduate practitioners entering the workforce and once practising, the majority enter private practice in the face of limited public health employment opportunities.

## Background

All health practitioners who practise in a regulated profession in New Zealand (NZ) must be registered with the relevant responsible authority and hold an Annual Practising Certificate (APC) issued by that authority. To practise as a podiatrist in NZ the health professional must be registered and hold an APC issued by the Podiatrists Board of New Zealand (PBNZ). Podiatry is one of 17 health professions in NZ regulated under the Health Practitioners Competence Assurance (HPCA) Act 2003 [1]. Since 2014, as part of the application process, all podiatrists who apply for their APC complete an online workforce survey (the survey) that solicits basic workforce characteristics. This study aimed to generate a profile of the NZ podiatry profession through examining the survey data from 2015 to 2019.

### Brief history of podiatry in NZ

Podiatry (formerly called chiropody) first became a registered profession in NZ in 1967 through the Chiropodists Regulations (1967) under the Medical and Dental Auxiliaries Act 1966 [2]. Chiropody was defined as “the prevention or treatment for fee or reward of any painful or deforming condition of the human foot by physical means other than means which should be used only by a medical practitioner” [2]. Under these regulations chiropody could be performed by a medical practitioner, an assistant of a medical practitioner, a registered chiropodist, any person acting under the control of a medical practitioner or chiropodist, any person as part of a course of training for students seeking to qualify as medical practitioners or chiropodists, a registered physiotherapist, and anybody performing in the capacity of district nursing [2]. The terms ‘podiatry’ and ‘podiatrist’ came into effect in 1982 under the Podiatrists Regulations (1982) [3]. However, the definition of podiatry remained unchanged from that of chiropody. In 2004 with the implementation of the HPCA Act (2003) the term ‘podiatrist’ became protected and four named scopes of practice were introduced [1]. The PBNZ assumed full responsibilities for the named scopes of practice being Podiatrist, Podiatric Surgeon, Podiatric Radiographic Imager and Podiatric Prescriber. A podiatrist is defined as “a registered primary health care practitioner (including those previously registered as a chiropodist) who utilises medical, physical, palliative and surgical means other than those prescribed in the podiatric surgeon scope of practice, to provide diagnostic, preventative and rehabilitative treatment of conditions affecting the feet and lower limbs”. In 2006 an additional scope of practice, Visiting Podiatrist Educator was added. In 2011 the Scope of Podiatric Prescriber was revoked, leaving four scopes of practice in which NZ podiatrists can now practice. Currently, the Bachelor of Health Science (Podiatry) which is delivered by Auckland University of Technology (AUT) is the only qualification delivered in NZ which leads to registration with the PBNZ.

## Methods

Between the years of 2015 to 2019 all NZ registered podiatrists completed a 14-question online survey as part of their annual application for an APC. An adapted version of the online survey is presented in Additional File 1. The survey contained questions related to scope of practice, employment status, main work setting, work type in main setting, work location, professional memberships, professional qualifications, availability of peer support, and ethnicity. The work location question asked practitioners to name their location by geographical locality to their nearest District Health Board (DHB). In NZ there are 20 DHBs that are responsible for providing or funding the provision of health services including public hospitals. All survey data is held by the PBNZ in a protected database.

Survey data was downloaded by a second party who provide data security for the PBNZ database. The data provided for analysis had all identifiers removed so that data could not be re-linked to the individual practitioner. Additional information sought from the PBNZ related to the number of graduating students from AUT who applied for registration and were issued an APC. Consultation was undertaken with the AUT Ethics Committee (AUTEC) and it was determined that ethical approval was not required for the study.

All data was provided by PBNZ in an Excel spreadsheet separated into six categories: demographics, main work setting, work type in main setting, employment status, work location, work hours, and NZ graduates entering practice. Survey responses from 2015 to 2019 relating to work setting, employment status, work hours, location, and number of graduates entering practice were analysed. Workplace setting data were analysed by means and percentages and assessment of mean annual percentage growth. Work hours data were analysed by means and percentages and assessment of the 5-year mean. Data for work type, employment status, work location and the number of graduates entering practice were analysed by assessment of the means and percentages. The questions relating to ethnicity and location have only been included in the survey since 2018, consequently ethnicity and location data were only included for the years 2018 and 2019.

## Results

As of 31 March 2019 there were 430 registered podiatrists in NZ. Fifty three percent (*n* = 228) declared themselves as NZ European, 6% Māori (*n* = 24) and 5% British/Irish (*n* = 23). All other declared nationalities represented less than 5% of the profession. Sixty-seven percent (*n* = 288) were female and 33% (*n* = 148) were male.

### Main workplace setting

A summary of the main workplace setting of NZ podiatrists is presented in Table 1. Over the study period (2015 to 2019) the total number of podiatrists with APCs increased by approximately 20% (*n* = 73), with a mean annual growth of 4.1%. The number of podiatrists working in private practice increased by 36% (*n* = 253 to *n* = 345), with a mean annual growth of 7.2%. The number of podiatrists working in DHBs increased by 44% (n = 25 to *n* = 36), mean annual growth 8.8%. The mean percentage of podiatrists working in DHBs remained constant with a mean annual growth of 8.8%. The mean percentage of podiatrists working in private hospital (4%) and university settings (2%) remained small and stable over the study period.

**Table 1:** Main workplace setting of podiatrists who held an Annual Practising Certificate 2015 to 2019

Workplace setting	2015	2016	2017	2018	2019	Mean annual growth (%)
Private Practice, n (%)	253 (71)	290 (77)	305 (79)	325 (80)	345 (80)	7
DHB – Hospital, n (%)	25 (7)	28 (7)	28 (7)	33 (8)	36 (8)	9
Private Hospital/Rest Home, n (%)	18 (5)	19 (5)	23 (6)	22 (6)	17 (4)	−1
University, n (%)	10 (3)	10 (3)	10 (3)	10 (2)	7 (2)	−6
Other, n (%)	28 (8)	27 (7)	15 (3)	10 (2)	15 (3)	**NA**
No response, n (%)	23 (6)	5 (1)	7 (2)	6 (2)	10 (3)	**NA**
**Total APC holders**	**357**	**379**	**388**	**406**	**430**	**4.1**

APC, annual practising certificate; DHB, District Health Board

### Work type in main practice setting

Table 2 shows the work type undertaken by podiatrists in their main practice setting. The most common work type reported across the entire study period was general podiatry. Diabetes podiatry and sports medicine were reported as the next most common, with the trends amongst these work types remaining relatively constant over the study period.

**Table 2:** Work type in main setting of podiatrists who held an Annual Practising Certificate 2015 to 2019

Work type	2015	2016	2017	2018	2019
General podiatry, n (%)	280 (35)	321 (34)	319 (33)	351 (31)	364 (30)
Diabetes podiatry, n (%)	180 (22)	211 (22)	220 (22)	250 (22)	260 (21)
Sports medicine, n (%)	153 (19)	170 (18)	172 (18)	207 (18)	234 (19)
Surgery, n (%)	71 (9)	93 (10)	107 (11)	136 (12)	147 (12)
Management, n (%)	57 (7)	76 (8)	78 (8)	109 (9)	119 (10)
Teaching, n (%)	23 (3)	37 (4)	41 (4)	43 (4)	43 (3)
Technical representative, n (%)	6 (1)	5 (1)	7 (1)	6 (1)	7 (1)
Other, n (%)	15 (2)	17 (2)	30 (3)	28 (2)	31 (3)
No response, n (%)	23 (2)	5 (1)	7 (1)	12 (1)	20 (1)
**Total responses**	**808**	**935**	**981**	**1142**	**1225**

### Employment status

Over the study period the trends within the employment categories remained constant (Table 3). The majority of podiatrists (69%) declared themselves as self-employed or business owners. Approximately a quarter of the profession were full-time employees with a smaller percentage (12%) declaring themselves as part-time employees.

**Table 3:** Employment status of podiatrists who held an Annual Practising Certificate 2015 to 2019

Employment status	2015	2016	2017	2018	2019
Self-employed, n (%)	134 (37)	143 (38)	156 (41)	172 (42)	172 (40)
Full time employee, n (%)	80 (22)	90 (23)	98 (25)	94 (23)	110 (25)
Business owner / director, n (%)	62 (17)	79 (21)	70 (18)	82 (20)	84 (20)
Part time employee, n (%)	34 (10)	50 (13)	40 (10)	38 (9)	54 (12)
Other, n (%)	20 (6)	15 (4)	22 (6)	17 (4)	8 (2)
No response, n (%)	27 (8)	2 (1)	0 (0)	7 (2)	6 (1)
**Total responses**	**357**	**379**	**386**	**410**	**434**

### Work location in NZ

In 2019 69% of practitioners were located in the North Island (*n* = 297) with 33% (*n* = 142) of the total podiatrists holding an APC located in the greater Auckland region. Forty three percent (*n* = 186) of all NZ podiatrists practiced in an area north of Hamilton. Of the practitioners in the South Island (*n* = 123) 45% (*n* = 55) were in the Canterbury region. Between 2018 and 2019, 46 new podiatrists started work in a new location, 67% (*n* = 31) in the North Island and 33% (*n* = 14) in the South Island. Of the new practitioners to the North Island 66% (*n* = 20) were in the Auckland and Hamilton region. The Wellington and Taranaki regions had no new podiatrists. In the South Island of the 15 new podiatrists who began work, 73% (*n* = 11) were in the Canterbury region. The 2018 and 2019 location data are displayed in Table 4.

**Table 4:** Location of podiatrists who held an Annual Practising Certificate 2018 to 2019

	2018	2019
**North Island**		
Northland, n (%)	13 (3)	12 (3)
Auckland, n (%)	72 (18)	79 (18)
Waitemata, n (%)	36 (9)	42 (10)
Counties Manukau, n (%)	18 (4)	21 (5)
Waikato, n (%)	28 (7)	32 (7)
Bay of Plenty, n (%)	24 (6)	26 (6)
Hawkes Bay, n (%)	13 (3)	15 (3)
Taranaki, n (%)	9 (2)	9 (2)
Wairarapa, n (%)	3 (1)	5 (1)
Whanganui, n (%)	9 (2)	11 (3)
Hutt, n (%)	16 (4)	20 (5)
Capital and Coast, n (%)	25 (6)	25 (6)
**South Island**		
Nelson, n (%)	11 (3)	10 (2)
Canterbury, n (%)	44 (11)	55 (13)
Southern, n (%)	27 (7)	31 (7)
No response, n (%)	58 (14)	37 (9)
**Total North Island podiatrists, n (%)**	**266 (66)**^**a**^	**297 (69)**^**a**^
**Total South Island podiatrists, n (%)**	**82 (20)**^**a**^	**123 (22)**^**a**^
**Total NZ podiatrists**	**406**	**430**

^**a**^percentage will not total 100 due to non-responses

### Work hours

The reported hours worked per week and per annum are displayed in Table 5. Over the study period 37% of podiatrists indicated they worked between 31 and 40 h per week with 23% indicating they worked greater than 40 h per week. Fifty two percent indicated they worked between 41 and 50 weeks per year. Forty four percent reported working 46 to 50 weeks per year.

**Table 5:** Work hours per week and per annum of podiatrists who held an Annual Practising Certificate 2015 to 2019

Hours worked per week	2015	2016	2017	2018	2019	5 year mean (%)
10 h or less per week, n (%)	22 (6)	26 (7)	25 (6)	20 (5)	16 (4)	6
11 to 20 h per week, n (%)	50 (14)	55 (14)	52 (13)	59 (15)	53 (12)	14
21 to 30 h per week, n (%)	58 (16)	58 (15)	50 (13)	60 (15)	71 (16)	15
31 to 40 h per week, n (%)	126 (35)	139 (37)	141 (37)	146 (36)	175 (41)	37
Over 40 h per week, n (%)	77 (22)	83 (22)	92 (24)	107 (26)	102 (24)	23
No response, n (%)	24 (7)	18 (5)	28 (7)	14 (3)	13 (3)	5
**Hours worked per annum**						
1 to 5 weeks, n (%)	6 (2)	3 (1)	7 (2)	7 (2)	18 (4)	2
6 to 10 weeks, n (%)	11 (3)	5 (1)	6 (2)	10 (3)	11 (3)	2
11 to 15 weeks, n (%)	9 (2)	16 (4)	13 (3)	14 (3)	6 (1)	3
16 to 20 weeks, n (%)	14 (4)	12 (3)	6 (2)	14 (3)	13 (3)	3
21 to 25 weeks, n (%)	1 (1)	4 (1)	5 (1)	3 (1)	11 (3)	1
26 to 30 weeks, n (%)	10 (3)	12 (3)	15 (4)	12 (3)	14 (3)	3
31 to 35 weeks, n (%)	4 (1)	6 (2)	5 (1)	13 (3)	13 (3)	2
36 to 40 weeks, n (%)	30 (8)	41 (11)	43 (11)	48 (12)	42 (10)	10
41 to 45 weeks, n (%)	42 (12)	47 (12)	46 (12)	47 (12)	45 (11)	12
46 to 50 weeks, n (%)	151 (42)	170 (45)	176 (45)	176 (43)	187 (43)	44
51 to 52 weeks, n (%)	41 (11)	42 (11)	43 (11)	44 (11)	49 (11)	11
No response, n (%)	38 (11)	21 (6)	23 (6)	18 (4)	21 (5)	6
**Total Practitioners**	**357**	**379**	**388**	**406**	**430**	

### New Zealand graduates entering practice

**Fig. 1:**
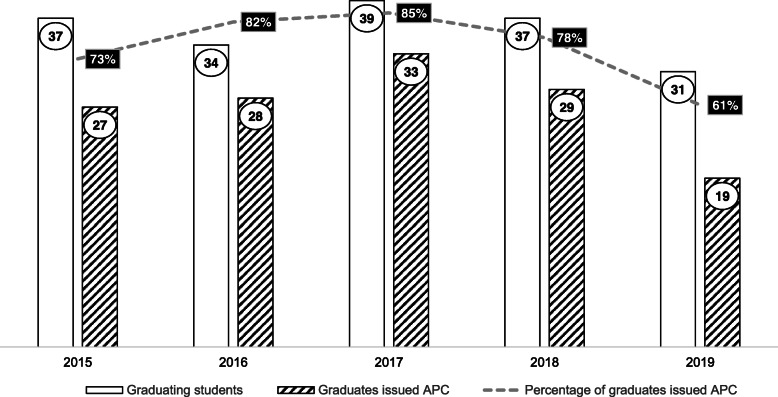
Number of graduates and percentage of graduates issued an APC

The number of graduating students with the number and percentage of graduates issued an APC in the year following their graduation are presented in Fig. 1. Over the study period there were 178 AUT graduates available to enter the NZ workforce. Between 2015 to 2019, 76% (*n* = 136) of graduates were issued an APC. The percentage of graduate conversions to APCs ranged from 61 to 85% over the study period, with 2019 recording the lowest number of graduate conversions to APC issue.

## Discussion

This is the first study to provide insight into the NZ podiatry profession through analysis of workforce data between 2015 to 2019. Although the profession has experienced modest growth during the study period the number of practising podiatrists in NZ remains relatively small. The data highlights concerning trends surrounding the current and future growth of the workforce. Areas of concern include the number of podiatrists currently practising, the limited number of new graduates entering the workforce, the limited opportunities for podiatrists to work in secondary care settings, and the small number of podiatrists working outside of major cities.

Whilst the number of NZ podiatrists grew by approximately 20% over the study period, the number of NZ podiatrists per capita remains small. The NZ population was estimated at 5.04 million in 2019, with 430 podiatrists holding an APC, this equates to approximately 8.5 podiatrists per 100,000 people. Comparably, Australian statistics for March 2019 indicate there were 5215 podiatrists registered in Australia, representing approximately 20 podiatrists per 100,000 people [4]. In the United Kingdom (UK), estimated figures are on par with Australian data for 2019, with approximately 20 podiatrists per 100,000 people. For NZ to achieve a per capita ratio equivalent to that of the UK and Australia (approximately 20/100,000) a total of 1008 practising podiatrists would have been required in 2019, an increase of 578 podiatrists on reported figures. With the knowledge that NZ has a lower per capita number of podiatrists we must also consider if the NZ population requires podiatrists or has a high podiatric need. To identify this, we can draw comparison to international data related to population types who commonly access podiatry services, namely, adults aged over 65 years old and people with diabetes [5–7]. Regarding adults aged 65 years and older, comparison between UK, Australian and NZ population estimates indicate similar rates between the three countries. The UK, 18.3% in 2019 [8], Australia,15.9% in 2019 [9] and NZ, 15.9% in 2020 [10]. Estimated diabetes prevalence rates are also similar between the countries. Prevalence rates in the UK were 6.2% in 2018 [11], in Australia, 4.9% in 2017–2018 [12] and in NZ, 5% in 2018 [13]. The lower per capita number of podiatrists in NZ, coupled with comparable international population estimates of people who commonly access podiatry, signals evidence of a NZ podiatry workforce shortage and subsequently a situation where the profession may not be meeting the podiatry needs of the population.

One solution to remedy the workforce issue is to increase the number of graduates entering the profession. However, NZ has only one tertiary provider of undergraduate podiatric education, and as shown in Fig. 1 only a small number of NZ graduates were available to enter the NZ workforce during the study period. Building on this issue is that post-graduation, not all graduates move on to enter NZ practice with only 61% of graduates issued an APC in 2019. Anecdotally the gap between students graduating and seeking registration can be explained by one of four scenarios. First, graduates seek registration to practise overseas and have no interaction with the PBNZ, therefore no data is recorded. Second, graduates move straight into another qualification that does not require registration. Third, graduates delay their registration until well into the year post-graduation. Finally, they decide not to pursue a career in podiatry. Unless graduate numbers significantly increase, the workforce shortage will only continue to escalate. Further evidence of a growing workforce shortage is revealed by examination of the relationship between total numbers of podiatrists leaving the profession and the number of new graduates entering the profession. Over the study period 136 new graduates entered the profession, however, this figure has not been reflected in the total number of podiatrists holding an APC which grew by only 73, indicating a loss of 63 practitioners within the NZ profession between 2015 to 2019. No data was available for analysis to provide insight into attrition in the NZ podiatry profession. We are unsure if this number represents a natural attrition or indicative of other factors such as professional dissatisfaction.

Although data indicated the number of podiatrists in private practice increased during the study period, this may be a situation driven out of necessity, due to the limited work opportunities for podiatrists, particularly in the public health sector. While data indicates there has been an increase in work for NZ podiatrists in the public health sector, there were only 32 podiatrists (8% of the 2019 practitioners) who declared themselves as employed in the public health sector. When compared to other countries this number is again low. The 2014 Australian workforce data indicated that 19.5% of podiatrists were working in a hospital or community healthcare service, with 65% employed in private practice [14]. UK workforce data from 2016 indicated that 35% of podiatrists worked in the National Health Service (NHS), 23% a mixture of NHS and private practice and 41% in private practice only [15]. These comparisons indicate there is a smaller percentage of the NZ podiatry workforce employed in the public health sector when compared to Australia and the UK. The smaller percentage of public sector work for NZ is multifactorial but warrants further exploration to understand barriers to recruitment into this sector of the workforce.

Further compounding the growing workforce issue is the small number of podiatrists located outside of the major cities. 2019 data indicated most practitioners were in the North Island with approximately half of those located in the Auckland region. This is not unexpected as the Auckland population accounted for approximately 40% of the North Island population in 2019. However, whilst Auckland is the most densely populated area, there is still a low per capita ratio of podiatrists in Auckland (approximately 10 podiatrists per 100,000 people). Data for the South Island also demonstrated a similar per capita ratio (approximately 10 podiatrists per 100,000 people) as in Auckland, however, with more podiatrists in the Auckland region than the entire South Island and 45% of South Island practitioners located in a similar geographical location (Canterbury) workforce concerns are raised. Concerns specifically related to the ability of podiatrists to service remote and rural communities.

The issues described by the survey data raise questions around the value, impact, profile, and sustainability of the NZ podiatry profession. With relatively few practising podiatrists and a slow rate of growth, the ability of the profession to demonstrate an impact on the health care system is limited. This is emphasised by the small number of podiatrists practising in the public health sector. Perhaps the greatest threat to the future of the profession is the small number of NZ graduates entering the workforce. Further work is required surrounding the visibility of the profession and whether the profile of podiatry must be lifted to attract and retain more people into the profession. The low profile raises questions as to whether there is an understanding of what a podiatrist does and the scope of work they can do; both from the perspective of potential students and from other health professionals. The effect of locality warrants further investigation. Specifically, we may be starting to see the long-term effects of having only one podiatry educational programme, which has been located in Auckland for 20 years. Furthermore, this North Island based programme may be limiting the ability to draw people from the South Island into the profession.

Although this is the first study to examine the NZ podiatry workforce, there are limitations that require consideration as the survey lacked the specificity to clearly articulate certain information. Two such examples include locality data which does not provide an accurate depiction of exact geographic location, as the question podiatrists were asked was to name their location in relation to their nearest DHB which can span a large geographical area. The second limitation, regarding work type data, was that it was difficult to accurately depict practising podiatrists exact work environment as the questionnaire had the ability to select multiple answers for the work type. This allowed those podiatrists holding multiple roles, for example simultaneous employment in academia, private practice and the public sector, to select multiple boxes. In addition to these two limitations, specificity was also affected by the fact that many of the questions in the survey did not require practitioners to respond. Consequently, there are numerous questions where there are non-responders.

## Conclusion

There is evidence of a NZ podiatry workforce shortage. Between the years of 2015 and 2019 the NZ podiatry profession underwent minimal growth with the number of podiatrists per capita less than half of Australia and the UK. To maintain a per-capita ratio of podiatrists approximate to Australia and UK, in 2019 NZ would have required an additional 578 podiatrists. There are not enough new practitioners entering the workforce. Once practising, the majority enter private practice with the numbers of private practitioners increasing in the face of limited public health employment opportunities.

## Supplementary information

**Additional file 1.** Workforce survey questions

## Data Availability

Request for further details of the data set and queries relating to data sharing arrangements may be submitted to Matthew Carroll (matthew.carroll@aut.ac.nz). The survey does not obtain consent for the participant data to be shared, although the present data are anonymised and with all personal identifiers removed.

## Abbreviations

AUT: Auckland University of Technology
AUTEC: Auckland University of Technology Ethics Committee
APC: Annual Practicing Certificate
DHB: District Health Board
HPCA Act (2003): Health Practitioners Competence Assurance Act (2003)
NZ: New Zealand
PBNZ: Podiatrist Board of New Zealand
UK: United Kingdom
