# Supplementary material for: The New Zealand podiatry profession – a workforce in crisis?
Source: J Foot Ankle Res. 2020 Oct 12;13:62. doi: 10.1186/s13047-020-00430-y (PMC7552341; doi:10.1186/s13047-020-00430-y)
Supplement: Supplementary file 1 — Additional file 1. Workforce survey questions [file 13047_2020_430_MOESM1_ESM.pdf]

The New Zealand podiatry profession – a workforce in crisis?
Additional File 1 – Workforce survey questions

(a) If you have answered “I am” to c, d or e, please provide the approximate number of weeks practised and the average number of hours per week practised in the last APC year:

Podiatry:______________________________ weeks for ____________ average hours per week

Podiatric Surgery: _______________________ weeks for ____________ average hours per week

Podiatric Radiographic Imagery: ___________ weeks for ____________ average hours per week

(b) **Are you currently** a

- ☐ Full time employee
- ☐ Part time employee
- ☐ Self- employed
- ☐ Business owner/ director
- ☐ Lecturer
- ☐ Other

(c) **Main workplace setting**

- ☐ Private practice
- ☐ DHB - hospital
- ☐ Private hospital/ rest home
- ☐ University
- ☐ Other

Second main workplace setting

- ☐ Private practice
- ☐ DHB - hospital
- ☐ Private hospital/ rest home
- ☐ University
- ☐ Other

(d) **Work type/s in main employment setting & average hours per week for each type**

- ☐ General podiatry hours
- ☐ Diabetes podiatry hours
- ☐ Sports medicine hours
- ☐ Surgery hours
- ☐ Teaching hours
- ☐ Technical representative hours
- ☐ Management hours
- ☐ Other hours

(e) **Name of main workplace**

(f) **Closest DHB (geographic area) to main place of employment**

(g) **In what other countries have you been registered to practice podiatry**

(h) **Postgraduate qualifications:**

- ☐ post grad certificate
- ☐ post grad diploma
- ☐ doctorate
- ☐ currently enrolled in post grad studies
- ☐ Masters
- ☐ Bachelors Honours
- ☐ Nil

(i) **I am a member of Podiatry NZ:** (Yes / No) Please cross one out

(j) **If no for above question, do you currently have professional indemnity insurance?** (Yes / No)

(k) **Are you a member of any other podiatry related professional group?**

- ☐ NZ Society for Study of Diabetes (Podiatrists Special Interest Group)
- ☐ NZ Wound Care Society
- ☐ Australasian Podiatry Council
- ☐ The Society of Chiropodists & Podiatrists
- ☐ PMPCPD Network
- ☐ British Chiropody & Podiatry Assoc
- ☐ Nil
- ☐ Other:

(l) **Peer support & supervision:** Please indicate in which option/s you are currently participating:

- ☐ Formal supervision (clinical/cultural)
- ☐ Formal clinical peer reviews
- ☐ Podiatry student placements (last 12 months)
- ☐ Regular podiatry based meetings (branch/regional/national)
- ☐ Nil
- ☐ Other:

(m) **Do you intend to continue to practice podiatry in New Zealand** for

- ☐ Less than 5 years
- ☐ Over 5 years but less than 10 years
- ☐ Over 10 years
- ☐ Unsure

(n) **Origin / Ethnicity**

Place of origin:

Ethnicity: *Please tick all relevant box/es*

- ☐ NZ European
- ☐ Māori
- ☐ Pasifika*
- ☐ Chinese
- ☐ Korean
- ☐ Indian
- ☐ Other European
- ☐ South East Asian
- ☐ Other

* Pasifika - indigenous people of the Pacific Islands
